# HIV Status Disclosure to Sexual Partners, among People Living with HIV and AIDS on Antiretroviral Therapy at Sokodé Regional Hospital, Togo

**DOI:** 10.1371/journal.pone.0118157

**Published:** 2015-02-06

**Authors:** Issifou Yaya, Bayaki Saka, Dadja Essoya Landoh, P’Niwè Massoubayo Patchali, Akouda Akessiwè Patassi, Abdoul-samadou Aboubakari, Makawa-Sy Makawa, Mathias Kouamé N’Dri, Sékandé Senanou, Bassan Lamboni, Daoudou Idrissou, Kao Tanang Salaka, Palokinam Pitché

**Affiliations:** 1 Laboratoire de Santé Publique (EA 3279), Aix-Marseille Université, Marseille, France; 2 Service de dermatologie et IST, CHU Sylvanus Olympio, Université de Lomé, Lomé, Togo; 3 Division de l’épidémiologie, Ministère de la santé, Lomé, Togo; 4 Centre Hospitalier Régional (CHR) de Sokodé, Service de dispensation d’antirétroviraux (ARV), Sokodé, Togo; 5 Service de maladies infectieuses, CHU Sylvanus Olympio, Université de Lomé, Lomé, Togo; 6 Service de gynéco-obstétrique, CHU- Kara, Kara, Togo; 7 Direction préfectorale de la santé de Tône, Dapaong, Togo; 8 Programme National de lutte contre les maladies non transmissibles, Ministère de la Santé, Lomé, Togo; 9 Division de la Santé Familiale, Ministère de la Santé, Lomé, Togo; 10 Division de la Santé Communautaire, Ministère de la Santé, Lomé, Togo; 11 Conseil National de Lutte contre les IST/VIH/Sida, Lomé, Togo; Brighton and Sussex Medical School, UNITED KINGDOM

## Abstract

**Background:**

Many studies have reported factors associated with HIV status disclosure among People Living With HIV and AIDS (PLWHA) but very few were conducted among PLWHA receiving ART. In Togo, no study on HIV status disclosure to sexual partners has been conducted among PLWHA on ART yet. We sought to document factors associated with HIV status disclosure among PLWHA receiving ART at Sokodé regional hospital in Togo.

**Method:**

This was a cross-sectional study conducted from May to July 2013 at the regional hospital of Sokodé among 291 PLWHA who had been on ART for at least three months.

**Results:**

A total of 291 PLWHA on ART were enrolled in this study. Their mean age (±SD) was 37.3±9.3 years and the sex ratio (Male/Female) was 0.4. Among them, 215 (74.6%) completed the questionnaire on HIV sero-status disclosure. We found that 131 PLWHA (60.9%) had disclosed their HIV sero-status to their sexual partners; 130 (60.5%) were aware of the HIV status of their sexual partners. In the multivariate analysis, the factors associated with HIV status disclosure to sexual partners were: adherence to ART (aOR = 4.89; 95%CI = [1.52; 15.78]), sexual partner awareness of HIV sero-status (aOR = 52.73; 95%CI = [14.76; 188.36]) and marital status of PLWHA (aOR = 6.10; 95%CI = [1.74; 21.37]).

**Conclusion:**

This study allowed us to note that the disclosure of HIV status to sexual partners is relatively low and to document the associated factors such as adherence to ART, sexual partner awareness of HIV sero-status and marital status.

## Background

In late 2012, it was estimated that the number of People Living with HIV and AIDS (PLWHA) worldwide was 35.3 million, of whom 71% were living in sub-Saharan Africa where three—quarters of all AIDS-related deaths occurred in 2012 [1]. In Togo, there were 130,000 PLWHA of whom more than 31,638 were receiving ART and about 5% of them lived in the Central region [2].

In most AIDS programs, strategies for the prevention of HIV transmission are mainly based on campaigns to raise public awareness and to incite them to change their individual behaviors in a supportive environment. Among these prevention strategies, the recommendations relating to HIV status disclosure hold a very important place [3,4,5]. Sharing HIV status information with sexual partner helps to create a relationship in which the HIV-positive person is likely to get psychological, moral or financial support from his/her partner in order to cope with the difficulties associated with HIV infection [6,7]. It also encourages the adoption of safer sexual behavior in PLWHA and may reduce the risk of HIV sexual transmission to the partner [8,9]. It has been shown that after disclosing HIV status to one’s sexual partner or a family member, the resulting social support constitutes a key factor in fostering and maintaining ART adherence [10]. However, the disclosure of one’s HIV status could result in negative consequences including the loss of social support. Many PLWHA, after disclosing their HIV status, are victims of discrimination, stigmatization, rejection and sometimes violent reactions [4,11]. Disclosing HIV status to a trusted person in the circle becomes a major psychological challenge faced by PLWHA, which AIDS programs are unremittingly trying to take up.

Although HIV status disclosure seems to be a very complex and difficult process, it is well documented that its prevalence remains high among PLWHA in sub-Saharan Africa and may reach 72.1% in West Africa (Mali and Burkina Faso) [12] and 95% in Uganda [13].

Many studies on the factors associated with HIV status disclosure among PLWHA have been conducted [6,7,8,11,12,13,14,15,16]. Most of them have reported that the level and outcomes of HIV status disclosure among PLWHA were influenced mostly by ART adherence and knowledge of the partner’s HIV status [17], the level of education, the economical status and the patient’s age [14], the use of ART as well as living in couple [7].

However, few of these studies were conducted among PLWHA receiving antiretroviral therapy [11,12,17]. In addition, no study on HIV status disclosure to sexual partners has been conducted among PLWHA on ART in Togo yet. We sought to determine the level of HIV status disclosure to sexual partners and to document the factors associated with HIV status disclosure among PLWHA receiving ART at Sokodé regional hospital in Togo.

## Method

### Study Design

This was a cross-sectional study conducted at Sokodé regional hospital from May to July 2013 among PLWHA who had been on ART for at least three months.

### Setting

Sokodé regional hospital is a health reference center of the central region which is one of the six health regions of Togo. The hospital is about 350 km away from the capital Lomé. It served four health districts with a total population of 654,074 inhabitants in 2013 [18]. Around 45% of the 1869 PLWHA registered in the central region are followed up in this hospital [2].

### Study Population and Sampling

**Fig 1:**
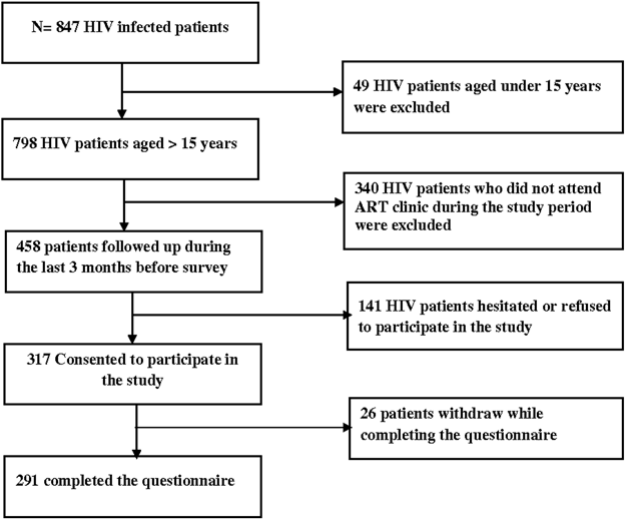
Flow chart of patients included in the study.

This study targeted PLWHA who were followed up at Sokodé regional hospital and receiving ART. Out of the 843 PLWHA who were being followed up in the hospital, 798 were aged above 15 years [2]. We used convenience sampling to recruit PLWHA aged above 15 years from the central health region who had been receiving ART for at least 3 months. Out of the 458 PLWHA on ART whom we met in the hospital, 317 consented to participate in the study and 291 PLWHA completed the interview (Fig. 1). HIV infected pregnant women were excluded from this study.

### Data Collection

Data were collected using a standardized pre-tested questionnaire in French, often explained in the local language. The questionnaires were filled in a private room at the ART clinic by the care givers during the appointment for ART renewal. Care givers were trained on the use of data collection tool to guarantee good understanding of the questionnaire. The questionnaire included socio-demographic information, clinical features, information on adherence to ART, information on HIV / AIDS knowledge and sexual behavior aspects. Data on HIV status disclosure to the regular sexual partners were collected only among sexually active PLWHA. Sexual activity was defined as reporting sexual intercourse with at least one sexual partner during the previous 3 months.

### Measurement

To measure adherence to treatment, two methods were used, including: i) timely attendance at appointments for delivery of antiretroviral drugs, here expressed as the number of appointments honored during the three months preceding the survey. Patients who did not fail any appointment were classified as adherent. ii) Counting the remaining tablets: The caregiver records the number of tablets or capsules remaining and evaluates adherence by considering a missed tablet or capsule as a tablet or capsule absorbed. Patients with a percentage of intake tablets or capsules greater than or equal to 95% were considered adherent.

The global index of adherence was obtained by summing the results of the two measurement of adherence methods used in the study (a coefficient assigned to each item with 0 for non adherence and 1 for adherence). Thus the global index of adherence was used to classify PLWHA in two categories according to the level of adherence to ART: non adherence (index from 0 to 1), good adherence (index = 2).

### Data Analysis

Data entry was performed using Epi Data software version 3.1. These data were then exported to SPSS Inc software Version 17.0 (SPSS Inc, Chicago, IL, USA) through which the statistical analyzes were performed.

For continuous variables, the mean and standard deviation were calculated while for categorical variables, we calculated proportions. Pearson chi-square test or Fisher′s exact test were used when appropriate in bivariate analysis. Our main outcome variable was PLWHA who had disclosed their HIV status to their partners.

Multivariate backwards stepwise logistic regression analysis was performed to identify independent risk factors associated with the main outcome. For this analysis, all significant variables were introduced in a logistic regression model to appreciate the adjusted effect and derive adjusted odds ratio (aOR) of each of the dichotomous dependent variables. A 95% level of confidence was applied throughout.

### Ethical Issues

This study was approved by the National AIDS and STI Program of Togo **(Ref N° 098/2013/MS/DSSP/PNLS-IST)**. An informed consent was signed by the participant after the verbal explanation. For patients under 18 years old (minors) who were enrolled in this study, an informed consent form was signed by their parents or guardians.

## Results

Out of the 317 PLWHA who consented to participate in the study, 291 completed the questionnaire. Among them, 90 PLWHA were men (30.9%). The mean (±standard deviation) age of participants was 37.3±9.3 years, 67% were living with their partners and 72.2% were in school.

Among the 291 PLWHA, 132 (45.4%) had been undergoing ART for more than three years, 106 (36.4%) were at WHO stage III or IV at the time of the survey and 218 (74.9%) had good adherence to ART (Table 1).

**Table 1:** Socio-demographic and clinical characteristics of PLWHA on ART at Sokodé regional hospital, Togo, 2013.

Patients characteristics	Total N = 291 (%)	HIV sero-status disclosure n = 215		OR	95%CI	p-value
		Yes **n(%)**	No n(%)			
**Age**						
Under 25 years	26 (8.9)	6 (42.9)	8 (57.1)	1	-	0.354
25–35 years	109 (37.5)	54 (62.8)	32 (37.2)	2.25	[0.72; 7.07]	
Over 35 years	156 (53.6)	71 (61.7)	44 (38.3)	2.15	[0.70; 6.62]	
**Gender**						
Male	90 (30.9)	54 (65.1)	29 (34.9)	1.33	[0.75; 2.35]	0.325
Female	201 (69.1)	77 (58.3)	55 (41.7)			
**Marital status**						
Married/ in couple	195 (67.0)	123(76.9)	37 (23.1)	19.53	[8.48; 45.01]	≤0,001
Single/widower/divorcee	96 (33.0)	8 (14.5)	47 (85.5)			
**Education level**						
Non schooled	81 (27.8)	34 (64.2)	19 (53.8)	1	-	0.073
Primary school	105 (36.1)	42 (52.5)	38 (47.5)	0.62	[0.30; 1.26]	
Secondary school	84 (28.9)	39 (61.9)	24 (38.1)	0.91	[0.43; 1.94]	
*High* school	21 (7.2)	16 (84.2)	3 (15.8)	2.98	[0.77; 11.55]	
**Location**						
Rural	97 (33.3)	51 (73.9)	18 (26.1)	2.34	[1.25; 4.38]	0.007
Urban	194 (66.7)	80 (54.8)	66 (45.2)			
**Knowledge of HIV sero-status of sexual partner**						
Yes	130 (60.5)	118 (90.8)	12 (9.2)	54.46	[23.57; 125.85]	≤0.001
No	85 (39.5)	13 (15.3)	72 (84.7)			
**WHO clinical stage**						
Stage I	53 (18.2)	25 (69.4)	11 (30.4)	1	-	0.011
Stage II	132 (45.4)	46 (49.5)	47 (50.5)	0.43	[0.19; 0.96]	
Stage III and IV	106 (36.4)	60 (69.8)	26 (30.2)	1.02	[0.44; 2.37]	
**Duration of ART**						
<1 year	33 (11.3)	6 (33.3)	12 (66.7)	1	-	0.033
1 to3 years	126 (43.3)	54 (60.7)	35 (39.3)	3.09	[1.06; 8.99]	
> 3years	132 (45.4)	71 (65.7)	37 (34.3)	3.84	[1.33; 11.06]	
**Adherence to ART**						
Yes	218 (74.9)	117 (69.2)	52 (30.8)	5.14	[2.53; 10.44]	≤0.001
No	73 (25.1)	14 (30.4)	32 (69.6)			
**Sero-status disclosure to regular partner**						
Yes	131 (60.9)	-	-	-	-	-
No	84 (39.1)					

Out of the 291 PLWHA included in the study, 215 (73.9%) answered the question on HIV disclosure status; among them, 131 (60.9%) had disclosed their HIV status to their sexual partners. Moreover, 130 PLWHA (60.5%) were aware of the HIV status of their sexual partners (Table 1).

During bivariate analysis, those who had disclosed their HIV status to their partners compared to those who had not, were more likely to be married or in couple (OR = 19.53; 95%CI = [8.48; 45.01]), to live in rural areas (OR = 2.34; 95%CI = [8.48; 45.01]), to be on ART for more than one year (p = 0.033), to know the HIV sero-status of their sexual partners (OR = 54.46; 95%CI = [23.57; 125.85]) and to have good adherence to ART (OR = 5.14; 95%CI = [2.53; 10.44]) (Table 1).

In multivariate analysis, three factors remained significantly associated with HIV sero-status disclosure: good adherence to ART (aOR = 4.89; 95%CI = 95%CI = [1.52; 15.78]), knowledge of the HIV status of sexual partners (aOR = 52.73; 95%CI = [14.76; 188.36]) and living in couple/married (aOR = 6.10; 95%CI = [1.74; 21.37) (Table 2).

**Table 2:** Multivariable analysis of HIV sero-status disclosure among PLWHA on ART at Sokodé regional hospital, Togo.

Patients characteristics	aOR	95% CI
**Marital status**		
Single/widower/divorcee	Ref	
Married /in couple	6.1	[1.74; 21.37]
**Location**		
Urban	Ref	
Rural	2.72	[0.84; 8.80]
**Knowledge of HIV sero-status of sexual partner**		
No	Ref	
Yes	52.73	[14.76; 188.36]
**WHO clinical stage**		
Stage I	Ref	
Stage II	0.35	[0.08; 1.53]
Stage III and IV	4.55	[0.94; 22.05]
**Duration of ART**		
Less than one year	Ref	
1 to 3 years	2.6	[0.50; 13.47]
More than 3 years	4.44	[0.93; 21.09]
**Adherence to ART**		
No	Ref	
Yes	4.89	[1.52; 15.78]

***aOR = adjusted odds ratio***

## Discussion

This study focused on the analysis of the factors associated with HIV sero-status disclosure to sexual partners among PLWHA on ART who are being followed at the regional hospital of Sokodé. The proportion of PLWHA who had disclosed their HIV status to their sexual partners was 60.9% in our study which is relatively low. Our findings are comparable to those reported by studies conducted in Sub-Saharan African countries: 50.9% in Uganda [15], 70% in Zimbabwe [19] or 72.1% in West Africa (Mali and Burkina Faso) [12].

However, high HIV disclosure rates were also reported in several studies. In the studies conducted in Ethiopia, Deribe *et al*. [8] and Seid *et al*. [11] reported respectively that approximately 90.8% and 93.1% of PLWHA who were interviewed had disclosed their HIV sero-status to their sexual partners. Likewise, Kouanda *et al*. [7] in Burkina Faso, Yonah *et al*. [20] in Tanzania and Ssali *et al*. [13] in Uganda observed respectively in their studies that, 81.4%, 93.3% and 95% of participants had disclosed their HIV sero-status to at least one person who is more likely to be their family member.

Despite the benefits of health care services offered to PLWHA in the regional hospital of Sokodé and social support activities implemented by non-governmental organizations, the proportion of PLWHA who did not disclose their HIV sero-status remains high (39.1%). This situation reflects the extent to which HIV remains a myth among populations [21] and the fear of negative reactions from neighbors such as the rejection, or physical abuse from partners. The silence on their HIV sero-status is most often accompanied by the adoption of sexual risk behavior leading to HIV transmission [8,16]. The explanation for this is to hide their health condition to others. Hence there is a need to strengthen the capacity of health care services to lead the majority of the PLWHA to disclose their HIV status to at least one person they rely on.

In a multivariate analysis, three factors were identified as being statistically associated with HIV sero-status disclosure. In our study, patients with good adherence to ART were 5 times more likely to inform their sexual partners about their HIV status. This is consistent with some findings of other studies that have demonstrated the positive effect of ART initiation on HIV sero-status disclosure [7,22]. It has been shown that patients who are adherent to ART are also those that are most likely to be regular in health care services [7,8]. Therefore, during their follow-up visit, they often receive other information and encouragement from care givers enabling them, not only to keep their ART, but also to create favorable psychological conditions to disclose their HIV status to their sexual partners. This study also reveals that patients on ART who know the HIV status of their sexual partners, either positive or negative, were more likely to disclose their HIV sero-status. This result is consistent with those reported by several studies in sub-Saharan African countries [7,11,12,16,23]. This could be explained by the fact that patients who know the HIV status of their sexual partners engage in discussions on HIV screening test, thus allowing them to anticipate the reactions of their partners during HIV status disclosure. Furthermore, knowing the HIV status of sexual partners encourages PLWHA to disclose their HIV status in order to prevent secondary transmission of HIV and to strengthen psychological and sometimes material support.

Finally, in our study, compared to PLWHA living alone, patients living in couple were more likely to disclose their HIV sero-status to their sexual partners. Similar results were reported by King et al. [6] in Uganda, Deride et al. [8] in Ethiopia, Kouanda et al. [7] in Burkina Faso and Amoran [16] in Nigeria. The patients living in union already have a stable relationship with their partners; this creates an environment of mutual trust and support, and therefore facilitates the sharing of information such as HIV sero-status [7,16]. While this is consistent with findings reported from some sub-Saharan African countries [7,16], it will not be true in some other religious areas. In studies conducted in Senegal [5] and Tanzania [24], the authors reported the negative effect of polygamy on HIV sero-status disclosure that could increase the vulnerability of PLWHA and women. In our study, we did not analyze the effect of the type of relationship (monogamy or polygamy) on HIV status disclosure.

This study was subject to a number of limitations. First, the sample may not be representative of the whole country. Sexual behavior may differ substantially across Togo, which has a diversity of cultures and religions. Second, we relied on self-reported sexual behavior through an interview, which may underestimate proportion of HIV status disclosure. The definition of concepts such as sexual partner and adherence to ART can vary from one study to another. Finally, we did not collect information on the type of relationship (monogamy and polygamy).

Our study is cross-sectional and therefore does not demonstrate the temporality between the cause and the outcome. Further cohort studies should be carried out to prove the temporality of the adherence to ART and HIV sero-status disclosure.

## Conclusion

In our study, the rate of HIV sero-status disclosure to sexual partners was relatively low. It is influenced by three factors including adherence to ART, knowledge of HIV status of sexual partners and marital status, but these results should be interpreted with caution because they cannot be extrapolated to all PLWHA in Togo. However, there is a need to strengthen the support and actions of secondary prevention services and to encourage PLWHA to disclose their HIV sero-status to sexual partners.

## References

[1] UNAIDS (2013) Global Report: UNAIDS Report on the global AIDS epidemic 2013.. Geneva UNAIDS

[2] Programme National de Lutte contre le Sida et les IST au Togo (2013) Rapport d’activités 2012. Lomé: PNLS—Togo 99 p. www.pnls.tg.

[3] ONUSIDA (2001) L’épidémie de VIH/SIDA en parler ouvertement Principes directeurs pour la divulgation à des fins bénéfiques, le conseil au partenaire dans le respect de l’éthique, et l’emploi approprié de la déclaration des cas d’infection à VIH. Genève: ONUSIDA pp. 43.

[4] Kyaddondo D, Wanyenze RK, Kinsman J, Hardon A (2013) Disclosure of HIV status between parents and children in Uganda in the context of greater access to treatment. SAHARA J 10 Suppl 1: S37–45. 23844801 10.1080/02664763.2012.755323

[5] Sow K (2013) HIV disclosure in polygamous settings in Senegal. SAHARA J 10 Suppl 1: S28–36. 23844800 10.1080/02664763.2012.755322

[6] King R, Katuntu D, Lifshay J, Packel L, Batamwita R, et al (2008) Processes and outcomes of HIV sero-status disclosure to sexual partners among people living with HIV in Uganda. AIDS Behav 12: 232–243. 17828450 10.1007/s10461-007-9307-7

[7] Kouanda S, Yameogo WM, Berthe A, Bila B, Bocoum Yaya FK, et al (2012) [Self-disclosure of a HIV-positive sero-status: factors favoring disclosure and consequences for persons living with HIV/AIDS in Burkina Faso]. Rev Epidemiol Sante Publique 60: 221–228. 10.1016/j.respe.2011.12.135 22595419

[8] Deribe K, Woldemichael K, Wondafrash M, Haile A, Amberbir A (2008) Disclosure experience and associated factors among HIV positive men and women clinical service users in Southwest Ethiopia. BMC Public Health 8: 81 10.1186/1471-2458-8-81 18312653 PMC2275263

[9] Pinkerton SD, Galletly CL (2007) Reducing HIV transmission risk by increasing serostatus disclosure: a mathematical modeling analysis. AIDS Behav 11: 698–705. 17082982 10.1007/s10461-006-9187-2 PMC2408867

[10] Stirratt MJ, Remien RH, Smith A, Copeland OQ, Dolezal C, et al (2006) The role of HIV serostatus disclosure in antiretroviral medication adherence. AIDS Behav 10: 483–493. 16721505 10.1007/s10461-006-9106-6

[11] Seid M, Wasie B, Admassu M (2012) Disclosure of HIV positive result to a sexual partner among adult clinical service users in Kemissie district, northeast Ethiopia. Afr J Reprod Health 16: 97–104. 22783673

[12] Ndiaye C, Boileau C, Zunzunegui MV, Koala S, Aboubacrine SA, et al (2008) Gender-related factors influencing HIV serostatus disclosure in patients receiving HAART in West Africa. World Health Popul 10: 43–54. 19369822

[13] Ssali SN, Atuyambe L, Tumwine C, Segujja E, Nekesa N, et al (2010) Reasons for disclosure of HIV status by people living with HIV/AIDS and in HIV care in Uganda: an exploratory study. AIDS Patient Care STDS 24: 675–681. 10.1089/apc.2010.0062 20863244 PMC3826576

[14] Kiula ES, Damian DJ, Msuya SE (2013) Predictors of HIV serostatus disclosure to partners among HIV-positive pregnant women in Morogoro, Tanzania. BMC Public Health 13: 433 10.1186/1471-2458-13-433 23641927 PMC3668140

[15] Osinde MO, Kakaire O, Kaye DK (2012) Factors associated with disclosure of HIV serostatus to sexual partners of patients receiving HIV care in Kabale, Uganda. Int J Gynaecol Obstet 118: 61–64. 10.1016/j.ijgo.2012.02.008 22507263

[16] Amoran OE (2012) Predictors of disclosure of sero-status to sexual partners among people living with HIV/AIDS in Ogun State, Nigeria. Niger J Clin Pract 15: 385–390. 10.4103/1119-3077.104507 23238184

[17] Coutherut J, Desclaux A (2014) [Disclosing HIV status: the experience of PLHIV after 10 years of ARV treatment in Senegal.]. Bull Soc Pathol Exot. 10.1007/s13149-014-0343-z 24595887

[18] Ministère de la santé (2012) Plan national de développement sanitaire 2012–2015. Lomé: Ministère de la Santé.

[19] Kangwende RA, Chirenda J, Mudyiradima RF (2009) HIV status disclosure among people living with HIV/AIDS at FASO, Mutare, Zimbabwe. Cent Afr J Med 55: 1–7. 21977839 10.4314/cajm.v55i1-4.63632

[20] Yonah G, Fredrick F, Leyna G (2014) HIV serostatus disclosure among people living with HIV/AIDS in Mwanza, Tanzania. AIDS Res Ther 11: 5 10.1186/1742-6405-11-5 24450933 PMC3900936

[21] Roura M, Busza J, Wringe A, Mbata D, Urassa M, et al (2009) Barriers to sustaining antiretroviral treatment in Kisesa, Tanzania: a follow-up study to understand attrition from the antiretroviral program. AIDS Patient Care STDS 23: 203–210. 10.1089/apc.2008.0129 19866538 PMC2776987

[22] Erku TA, Megabiaw B, Wubshet M (2012) Predictors of HIV status disclosure to sexual partners among people living with HIV/AIDS in Ethiopia. Pan Afr Med J 13: 87 23396625 PMC3567411

[23] Simbayi LC, Kalichman SC, Strebel A, Cloete A, Henda N, et al (2007) Disclosure of HIV status to sex partners and sexual risk behaviours among HIV-positive men and women, Cape Town, South Africa. Sex Transm Infect 83: 29–34. 16790562 10.1136/sti.2006.019893 PMC2598581

[24] Antelman G, Smith Fawzi MC, Kaaya S, Mbwambo J, Msamanga GI, et al (2001) Predictors of HIV-1 serostatus disclosure: a prospective study among HIV-infected pregnant women in Dar es Salaam, Tanzania. AIDS 15: 1865–1874. 11579250 10.1097/00002030-200109280-00017 PMC6261328

